# Magnetotaxial Perpendicular Magnetic Anisotropy and Enhanced Faraday Rotation in Ion Beam Sputtered Cerium-Substituted Yttrium Iron Garnet

**DOI:** 10.1021/acsaom.5c00496

**Published:** 2025-11-25

**Authors:** Taichi Goto, Takumi Koguchi, Yuki Yoshihara, Hibiki Miyashita, Kanta Mori, Toshiaki Watanabe, Allison C. Kaczmarek, Pang Boey Lim, Mitsuteru Inoue, Caroline A. Ross, Kazushi Ishiyama

**Affiliations:** † Research Institute of Electrical Communication, 13101 Tohoku University, 2-1-1 Katahira, Aoba, Sendai, Miyagi 980-8577, Japan; ‡ Graduate School of Engineering, Tohoku University, 6-6 Aramaki, Aoba, Sendai, Miyagi 980-8579, Japan; § 13129 Toyohashi University of Technology, 1-1 Hibarigaoka, Tempaku, Toyohashi, Aichi 441-8580, Japan; ∥ 92049 Shin-Etsu Chemical Co., Ltd., 2-13-1 Isobe, Annaka, Gunma 379-0195, Japan; ⊥ 2167 Massachusetts Institute of Technology, 77 Massachusetts Avenue, Cambridge, Massachusetts 02139, United States

**Keywords:** magnetooptical effect, Faraday rotation, magnetic domain, epitaxial growth, micromagnetics

## Abstract

Ferrimagnetic iron garnets are valuable for photonic and spintronic devices because of their large magnetooptical (MO) effects and tunable magnetic anisotropy and domain structures. In particular, cerium-substituted yttrium iron garnet (Ce:YIG) has an excellent MO figure of merit in the near-infrared region. However, achieving perpendicular magnetic anisotropy (PMA) in Ce:YIG films has conventionally relied on strain engineering, constraining the relationship between substrate lattice parameters and magnetic properties. We demonstrate strain-independent PMA in epitaxially grown Ce:YIG (Ce_0.9_Y_2.1_Fe_5_O_12_) films on two different (111)-oriented garnet substrates using ion beam sputtering. Despite opposite strain states (tensile and compressive), both films exhibit robust PMA with labyrinth-shaped magnetic domains with widths of 219 nm. Comprehensive surface and interface characterization reveals high-quality epitaxial growth with coherent film–substrate interfaces. The films demonstrated superior MO performance with a Faraday rotation of −1.05°/μm and a figure of merit of 74.7°/dB at 1064 nm wavelength. Detailed anisotropy analysis reveals that magnetotaxial (growth-induced) anisotropy of up to ∼30 kJ/m^3^ dominates over magnetoelastic contributions, enabling strain-independent PMA formation. Three-dimensional (3D) micromagnetic simulations confirm mixed Néel–Bloch domain wall configurations. This work demonstrates that PMA in Ce:YIG films can be achieved through magnetotaxial anisotropy independent of the substrate strain state, providing valuable insights for magnetooptical material design.

## Graphical abstract

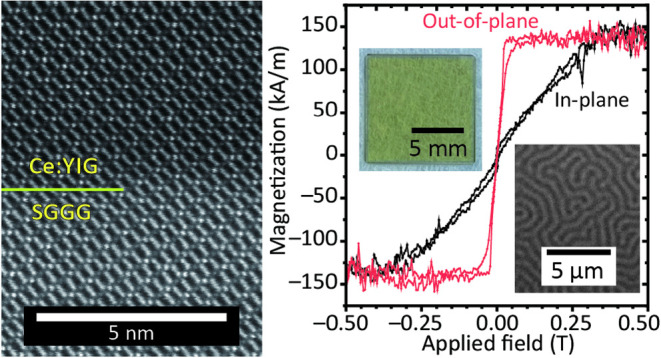

## 1 Introduction

Iron garnets, a class of ferrimagnetic materials, have garnered significant attention in photonics and spintronics due to their substantial magnetooptical (MO) properties,^1^ fast dynamics due to a low Gilbert damping,^2,3^ and their high-speed domain wall motion.^4^ Many compositions of iron garnet films have been studied in both single-crystalline and polycrystalline forms and have been applied in a wide range of devices. For instance, single-crystalline iron garnets have been used as optical isolators^5,6^ and Q-switches in high-power lasers;^7–9^ their large MO responses have been instrumental in observing spin dynamics including ferromagnetic resonance and spin waves;^10,11^ and they have been incorporated into current sensor applications.^12^ On the other hand, polycrystalline iron garnets deposited on silicon or glass substrates have been used in integrated optical isolators^13,14^ and spatial light modulators,^15,16^ including three-dimensional (3D) displays^17^ and holographic media.^18^ In many of these devices, a large MO effect and low absorption were the primary material requirements. However, in recently emerging applications such as Q-switches^7^ and 3D displays^17^ utilizing MO diffraction caused by phase interference phenomena in magnetic materials, as well as neural network elements^19^ and random number generators,^20^ the presence of small magnetic domains and perpendicular magnetic anisotropy (PMA) have become an additional critical requirement.

Cerium-substituted yttrium iron garnet (Ce:YIG) is an excellent candidate due to its large MO response in the near-infrared (NIR) region and its low absorption.^6,21–35^ Conventionally, achieving PMA in Ce:YIG films relies on strain engineering through careful lattice parameter matching between films and substrates. In ref 26, an ∼100-nm-thick single-crystalline Ce_1_Y_2_Fe_5_O_12_ film was grown on a (111)-oriented Gd_3_Ga_5_O_12_ (GGG) substrate using pulsed laser deposition (PLD), showing a smooth surface and an in-plane (IP) compressive strain state in the Ce:YIG film. However, the film had an IP magnetization with domain sizes exceeding ∼10 μm, as confirmed by MO Kerr effect (MOKE) microscopy at wavelengths of 406 and 635 nm. In this example, magnetoelastic (ME) anisotropy caused by the lattice constant mismatch between the substrate and the film was not sufficient to achieve PMA in Ce:YIG. On the other hand, Kuila et al. demonstrated PMA in 65-nm-thick Ce:YIG films prepared by PLD under different conditions^36^ and indicated a large contribution of growth-induced anisotropy,^37–43^ also known as magnetotaxial (MT) anisotropy,^37^ but their quantitative estimation was not performed because of the difficulty in separating ME and MT anisotropy contributions. In addition, the domain size was ∼100 μm, and the MO response was also reported as a relative value. The dominant role of ME anisotropy constrains the choice of substrate and limits applications. In this study, we demonstrate an approach to achieve PMA in Ce:YIG films that is independent of strain. We employed radio-frequency ion beam sputtering (RF-IBS) to grow Ce:YIG films on different garnet substrates with opposite strain states to investigate the contributions of ME and MT anisotropy and to establish that MT anisotropy can dominate over strain effects to achieve robust PMA independent of the film strain state.

## 2 Structural and Electronic Features

The Ce:YIG films were grown on (111)-oriented 10 mm × 10 mm gadolinium gallium garnet (GGG) and substituted gadolinium gallium garnet (SGGG) substrates in the same batch using an RF-IBS system with a base pressure of 4 × 10^–5^ Pa (Methods Section). The substrates were heated to 810 ± 30 °C according to previous reports^26,27,33,44^ and rotated at 4.3 rpm to ensure film uniformity. The value of ± 30 °C represents the maximum difference in temperature between the position on the sample holder where the temperature was measured, and the position on the sample holder where the substrate was mounted. The temperature variation during deposition was within ±1 °C. This deposition condition is the same as in our previous study on Ce:YIG growth on a yttrium aluminum garnet (YAG) substrate.^33^ The thickness of the Ce:YIG films was 129 ± 4.5 nm, measured by stylus profilometry. The error represents the standard deviation of nine repeated measurements. This thickness showed good agreement with that obtained by cross-sectional transmission electron microscopy (TEM, JEOL JEM-ARM200F). The composition of Ce:YIG was measured using an energy-dispersive spectrometer mounted on TEM, resulting in Ce_0.9±0.1_Y_2.1±0.1_Fe_5_O_12−δ_ where δ represents oxygen deficiency.

The crystal structure was characterized by using an X-ray diffractometer (XRD, Rigaku Smartlab), as shown in Figure 1. The ω–2θ scan around the (444) peaks of both Ce:YIG films showed clear Laue fringes (Figure 1a), indicating a smooth film surface and well-defined boundaries. No peaks other than those corresponding to the (*hhh*) directions were observed, consistent with epitaxial growth. Reciprocal space maps (RSMs) of the (336) planes were measured (Figure 1b,c). In both cases of GGG and SGGG substrates, the *q*_*x*_ values of the substrate peaks matched those of the Ce:YIG film peaks, indicating matching between the substrate and film IP lattice spacings. Based on a rhombohedrally strained unit cell, the lattice parameter *A*_CS_ and unit cell corner angle θ_CS_ of the films were estimated from the RSM results (Methods Section). The obtained values of films and substrates are shown in Table 1, close to those reported in previous studies.^26,34,45–47^ For comparison, the values for Ce:YIG films deposited on YAG substrates^33^ are also included. In the case of Ce:YIG/GGG, the value of *A*_CS_ was 1.2416 nm and that of θ_CS_ was 89.416°, indicating a tensile strain state in the out-of-plane (OP) direction and lattice mismatch of +0.38%. For Ce:YIG/SGGG, the value of *A*_CS_ was 1.2484 nm, and that of θ_CS_ was 90.179°, indicating a tensile strain state in the IP direction and lattice mismatch of −0.12%. Figure 1d,e shows the pole figures for the (444) peaks. One central peak at α = 0° and three peaks with 3-fold symmetry at α = 70.5° were observed, indicating epitaxial growth. The (640) peak resulting from deformation of the garnet structure was not detected. Compared to Ce:YIG/YAG, which exhibits a relaxed, defective, columnar structure due to the larger lattice mismatch,^33^ the Ce:YIG/GGG and Ce:YIG/SGGG are epitaxial, homogeneous, and of high crystal quality.

**Figure 1:**
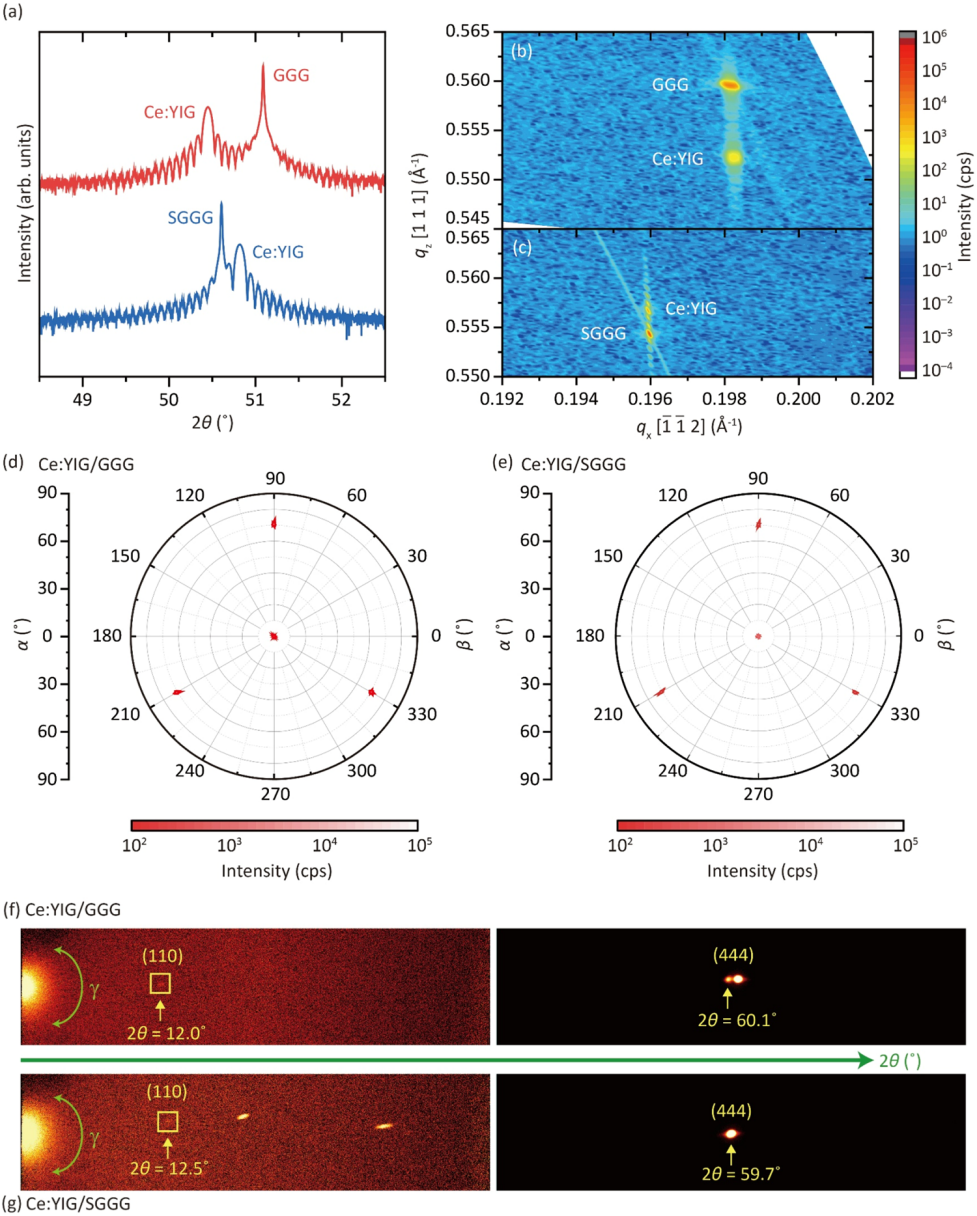
(a) XRD scan of the (444) peaks. No other peaks except the (444) and (888) of the film and substrate were observed. (b, c) RSM around the (336) peak for Ce:YIG/GGG and Ce:YIG/SGGG samples, respectively. (d, e) Pole figures at the (444) peak (2θ = 52.96°) for Ce:YIG/GGG and Ce:YIG/SGGG samples, respectively. (f, g) GADDS data for the (110) forbidden peaks and the (444) peaks of Ce:YIG/GGG and Ce:YIG/SGGG samples, respectively. The 2D maps were taken using a Co X-ray source for a range of 2θ and γ angles.

**Table 1:** Crystal Axis Length and Crystal Angle of the Deposited Ce:YIG and Substrates

Material	Crystal axis length, *A*_CS_ (nm)	Crystal angle, θ_CS_ ± 0.06° (°)	Tensile direction
Ce:YIG/GGG	1.2416	89.416	OP
Ce:YIG/SGGG	1.2484	90.179	IP
Ce:YIG/YAG	1.2434	89.838	OP
GGG	1.2369	89.942	–
SGGG	1.2499	90.004	–
YAG	1.2000	90.063	–

Figures 2 and S1 show cross-sectional high-angle annular dark-field (HAADF) images of Ce:YIG/GGG and Ce:YIG/SGGG along a [1̅01] zone axis, acquired by TEM (see the Methods Section). Images of the interface between the film and the substrate show coherent growth. Selected-area electron diffraction (SAD) patterns (Figure S2) show discrete spots, consistent with a single crystal. The overall high quality of the obtained Ce:YIG films is comparable to or superior to previous reports of films made using PLD and IBS.^26,48,49^

**Figure 2:**
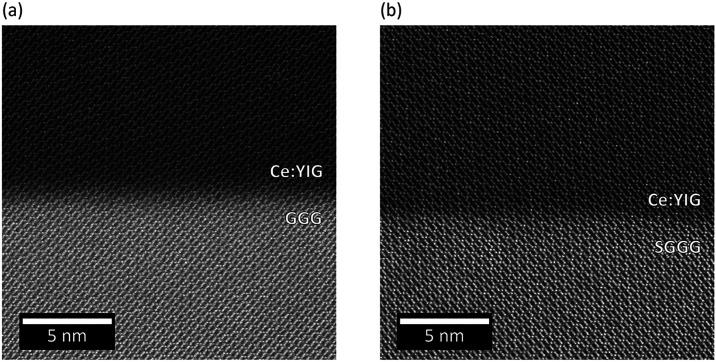
Cross-sectional HAADF TEM images of (a) Ce:YIG/GGG and (b) Ce:YIG/SGGG along the [1̅01] zone axis.

The oxidation of Ce^3+^ to Ce^4+^ is undesirable in Ce:YIG as it is accompanied by enhanced optical absorption and reduced magnetization and MO effects.^50^ X-ray photoelectron spectroscopy (XPS) was used to investigate the electronic states of the Ce:YIG films (Methods Section), obtaining a ratio of Ce^4+^ to Ce^3+^ from the Ce 3d peaks of 0.087 for both Ce:YIG/GGG and Ce:YIG/SGGG (Figure 3d). In comparison, Ce:YIG/YAG exhibited a ratio^33^ of 0.15, indicating a higher Ce^4+^ content than those of Ce:YIG/GGG and Ce:YIG/SGGG, possibly related to the higher defect levels. XPS probes only the near-surface region of a few nanometers. We cannot exclude a depth-dependent ratio, which could lead to depth-dependent magnetic, optical, and magnetooptical properties. The Fe LMM and Ce MNN regions observed in the Ce 3d and Fe 2p spectra correspond to the Auger electron peaks. The Fe 2p, Y 3d, and O 1s peaks are consistent with those reported elsewhere for Ce:YIG.^33,35,51^

**Figure 3:**
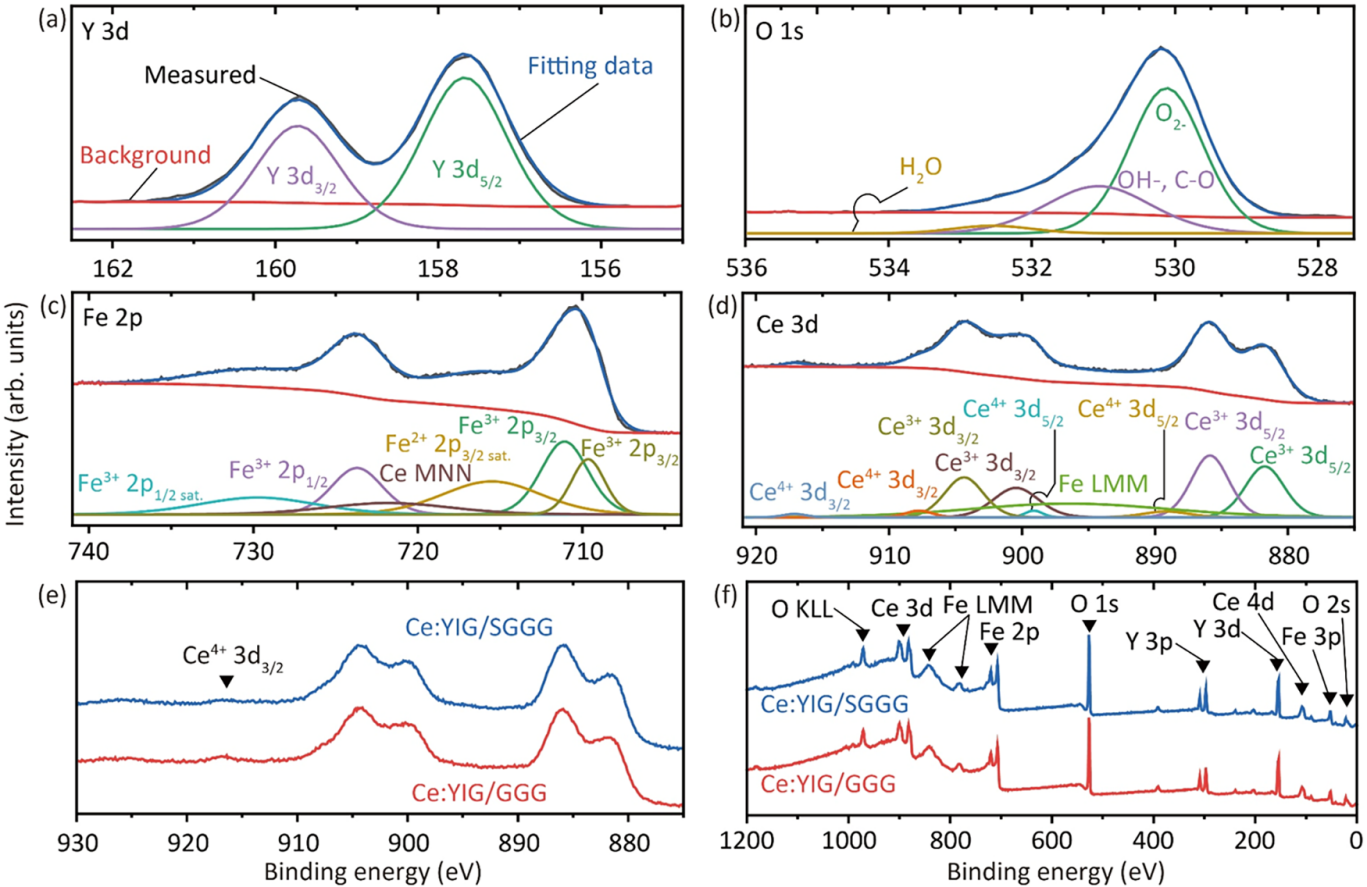
XPS spectra of Ce:YIG samples. (a) Y 3d, (b) O 1s, (c) Fe 2p, (d) Ce 3d, and (f) survey scan. Panels (a–d) show the spectra for Ce:YIG/GGG only, as Ce:YIG/SGGG exhibited almost identical results. (e, f) Spectra for both Ce:YIG/GGG and Ce:YIG/SGGG.

## 3 Magnetic Anisotropy Characteristics

Magnetic hysteresis loops of Ce:YIG/GGG and Ce:YIG/SGGG are shown in Figure 4, indicating that the films exhibit PMA and have saturation magnetization (*M*_s_) of 135 ± 5 and 138 ± 5 kA/m, respectively, consistent with previous reports.^21,26,33^ PMA implies that the magnetic easy axis is oriented normal to the film plane and represents the difference in energy between the magnetization lying OP versus the one lying IP. Hard axis loops were normalized to the saturation magnetization of the easy axis loops. The easy axis saturation field (*H*_s_) was approximately 70 mT for both films, and the IP and OP coercivities (*H*_c_) were 3 and 2 mT, respectively.

**Figure 4:**
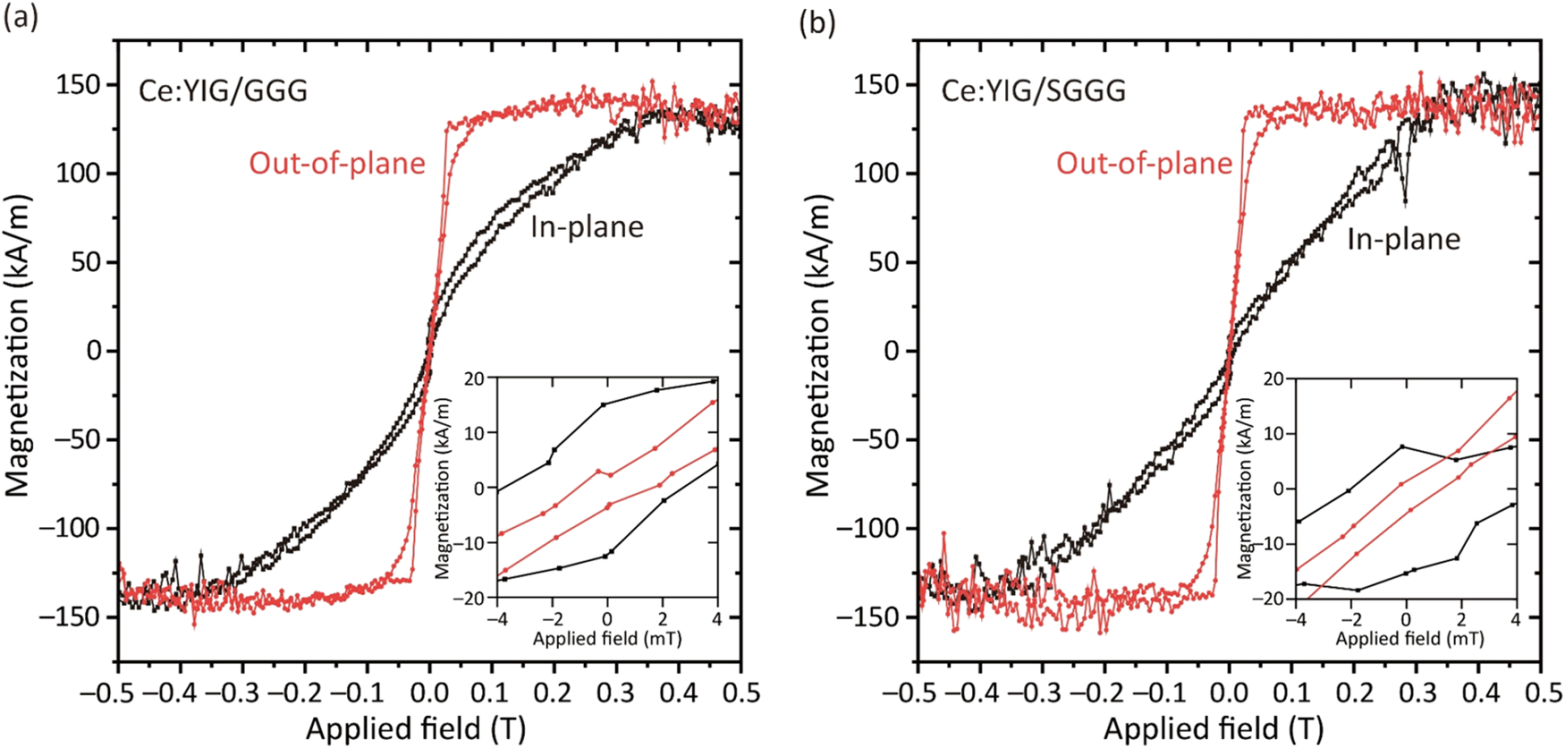
Magnetic properties of (a) Ce:YIG/GGG and (b) Ce:YIG/SGGG. The red plots show OP responses, and the black plots show IP responses. The insets show the loops near the zero field. The paramagnetic component was subtracted from the original data.

The magnetic anisotropy energy *K*_U_ was determined from the area difference between the IP and OP loops after subtracting the hysteresis by averaging the ascending and descending branches.^49^ *K*_U_ = *E*_IP_ – *E*_OP_ is the magnetic energy difference between IP (*E*_IP_) and OP (*E*_OP_) magnetization. The energy is *K*_U_ sin^2^ θ where θ is the angle from the OP direction, i.e., positive *K*_U_ indicates an OP easy axis.

The *K*_U_ values extracted from the hysteresis loops of Figure 4 were 14.9 ± 0.5 and 18.9 ± 0.7 kJ/m^3^ for Ce:YIG/GGG and Ce:YIG/SGGG, corresponding to anisotropy fields of μ_0_ *H*_A_ = 2*K*_U_/*M*_s_ = 221 ± 9 and 280 ± 11 mT. This value is 1 order of magnitude larger than that in a previous report, 7.8 kJ/m^3^.^36^ *K*_U_ is the sum of magnetocrystalline (MC) anisotropy (*K*_MC_), ME anisotropy (*K*_ME_), magnetostatic (MS) anisotropy (*K*_MS_), and MT anisotropy (*K*_MT_)­

1 KU=KMC+KME+KMS+KMT=−K112+94λ111c44(π2−θCS)−2πMs2+KMT

where λ_111_, *K*_1_, and *c*_44_ are the magnetostriction coefficient along [111], the first-order cubic MC anisotropy constant, and the shear modulus, respectively. For bulk YIG,^49,52^ *K*_1_ = −0.61 kJ/m^3^ (−6100 erg/cm^3^) and *c*_44_ = 76.6 GPa (7.66 × 10^11^ dyn/cm^2^). λ_111_ has been extrapolated from data of YIG^53^ and Ce:YIG^54^ with low Ce substitution according to λ_111_ = (17.7*x* −3) × 10^–6^ for Ce_*x*_Y_3–*x*_Fe_5_O_12_, yielding a value of +1.29 × 10^–5^ for Ce_0.9_Y_0.1_Fe_5_O_12_.^33^

We can determine *K*_ME_ + *K*_MT_ for each sample from the total anisotropy because *K*_MS_ is readily obtained from *M*_s_, and *K*_MC_ is small. The strain states of Ce:YIG/GGG and Ce:YIG/SGGG have opposite signs, implying that their *K*_ME_ values also differ. There are no reports of *K*_MT_ for Ce:YIG films, though *K*_MT_ has been investigated in other mixed-garnet films including Bi:YIG,^43^ TmIG,^38,39^ and EuTmIG^37^ films deposited by PLD, falling in the range of 1–10 kJ/m^3^.

The first scenario we consider assumes that both films have different *K*_MT_ values and the same literature value of λ_111_ = +1.29 × 10^–5^. This yields *K*_MT,GGG_ = 3.66 kJ/m^3^ and *K*_MT,SGGG_ = 37.2 kJ/m^3^, i.e., a strong dependence of *K*_MT_ on the lattice parameter of the substrate. Measurements of Bi:YIG grown at the same time on substrates with three different lattice parameters (GSGG, SGGG, and NGG) showed a large difference in *K*_MT_;^43^ hence, a substrate-dependent *K*_MT_ is not unreasonable for Ce:YIG. The derived *K*_MT_ values depend on the value of λ_111_, which is not well-known; for example, assuming instead λ_111_ = +0.65 × 10^–5^ (i.e., half the extrapolated value) yields 14.9 and 33.7 kJ/m^3^ for *K*_MT,GGG_ and *K*_MT,SGGG_, respectively.

In an alternative scenario, we assume the same *K*_MT_ for both films and allow λ_111_ to vary from the value extrapolated from the value reported in the literature. This yields λ_111_ = −1.73 × 10^–6^ and *K*_MT_ = 29.4 kJ/m^3^ (derivation steps are shown in Note S1). The negative λ_111_ is not consistent with the positive values expected from high Ce-content Ce:YIG, and it is also unlikely that *K*_MT_ would have no substrate dependence.

These results summarized in Table 2 indicate that *K*_MT_ is an important contribution to the anisotropy of Ce:YIG, with values on the order of 10 kJ/m^3^, an order of magnitude larger than that of *K*_ME_. These values fall within the higher range of *K*_MT_ values obtained from PLD mixed-garnet films,^47,48,53^ which may be related to the differences in growth kinetics of RF-IBS compared to PLD^55,56^ and/or the different dodecahedral species.

**Table 2:** Calculated Magnetic Anisotropy Energy

Scenario	Substrate	*M*_s_ (kA/m)	λ_111_	*K*_MC_ (kJ/m^3^)	*K*_ME_ (kJ/m^3^)	*K*_MS_ (kJ/m^3^)	*K*_MT_ (kJ/m^3^)	*K*_U_ (kJ/m^3^)	μ_*0*_ *H*_A_ (mT)
1	GGG	135	1.29 × 10^–5^	0.05	22.7	–11.5	3.66	14.9	221
	SGGG				–6.90		37.2	18.9	280
2	GGG	135	–1.73 × 10^–6^	0.05	–3.04	–11.5	29.4	14.9	221
	SGGG				0.92			18.9	280

To confirm the presence of cation ordering in the dodecahedral site that would contribute *K*_MT_, we used an XRD general area detector diffraction system (GADDS) to measure the {110} peaks, which would be absent in a perfect garnet lattice, following the procedure of ref 37. We see that both films exhibit a weak (110) peak, consistent with the ordering of Ce and Y in the dodecahedral sites. The (110) peak for Ce:YIG is predicted to be weak due to low contrast in electron densities of Ce and Y, in comparison to Bi:YIG.

## 4 Magnetooptical and Optical Responses

Faraday rotation (FR) and Faraday ellipticity (FE) spectra and loops were measured at 40 ± 0.5 °C using an MO measurement system (JASCO, J-1700FK, see the Methods Section). The FR spectra of Ce:YIG/GGG and Ce:YIG/SGGG are shown in Figure 5a. FR and FE values were defined as positive for clockwise rotation when viewed from the detector toward the source.^35,57^ The observed fringes result from thin-film interference. The spectra of both samples exhibited remarkable similarity, and their spectral features closely match those reported previously,^35^ characteristic of Ce substitution at Y sites. The inset of Figure 5a shows FR loops of Ce:YIG/GGG at wavelengths of 532, 1064, and 1550 nm. The FR values of Ce:YIG/GGG and Ce:YIG/SGGG were nearly identical (all MO data are shown in Figure S3). The FR values at these wavelengths for Ce:YIG/GGG were +1.75, −1.02, and −0.43°/μm. The FR of −1.02°/μm at a wavelength of 1064 nm is 1.6 times larger than the FR of −0.65°/μm exhibited by Ce:YIG/YAG,^33^ comparable to −1.07°/μm of polycrystalline Ce:YIG/silica, and 0.8 times that of Ce:YIG/GGG.^27^ These differences can be attributed to the Ce substitution ratio and population of Ce^3+^ ion states, as discussed in the XPS measurements. The coercivity of the FR loop was 2 ± 0.5 mT, and the required field to saturate the FR was 70 ± 10 mT due to the PMA of the films, significantly smaller than that in other reports: 200,^33^ 240,^35^ and 140 mT.^27^

**Figure 5:**
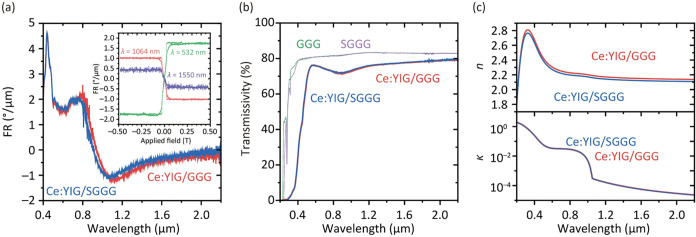
(a) FR spectra of Ce:YIG/GGG and Ce:YIG/SGGG. The inset shows FR loops measured at wavelengths of 532, 1064, and 1550 nm. (b) Transmittance spectra of Ce:YIG films on GGG and SGGG substrates and bare GGG and SGGG substrates. (c) Refractive index *n* and extinction coefficient κ spectra determined from the transmittance spectra.

Optical transmission spectra of the Ce:YIG/GGG, Ce:YIG/SGGG, GGG, and SGGG substrates were measured using a spectrophotometer (see the Methods Section), as shown in Figure 5b. Both Ce:YIG films exhibited similar transmission spectra, but Ce:YIG/SGGG showed slightly higher transmittance. The absorption edge wavelengths were 228 and 240 nm for Ce:YIG/GGG and Ce:YIG/SGGG, respectively. The high transmittance in the visible region is sufficient for practical device applications, with an estimated reflection coefficient of 14% at both the sample surface and film–substrate interfaces. Using these transmission spectra, the optical constants (refractive index *n* and extinction coefficient κ) were extracted through an analysis of interference fringes. The calculation was performed using fitting and simulation software (W. Theiss Hard- and Software, SCOUT version 3.04)^29^ implementing the Fresnel equation, a method validated by previous studies.^32,51,58^

The extinction coefficient κ shown in Figure 5c was converted to the absorption coefficient α_opt_ (dB/μm) using the relation α_opt_ = 10log_10_[exp­(−4πκ × 10^–6^/λ)]. The obtained α_opt_ values for Ce:YIG/GGG at wavelengths λ of 532, 1064, and 1550 nm were 2.24, 1.44 × 10^–2^, and 3.22 × 10^–3^ dB/μm, respectively. To evaluate the MO device performance, the figure of merit (FOM) defined as FR/α_opt_ was calculated for each wavelength λ (532, 1064, and 1550 nm), yielding values of 0.8, 70, and 132°/dB, respectively. The value at λ = 1064 nm shows a 1.4-fold improvement compared to the 50°/dB reported for Ce:YIG/YAG.^33^ These values are summarized in Table 3.

**Table 3:** Figure of Merit of the Deposited Ce:YIG/GGG Sample

λ (nm)	FR (°/μm)	κ	α_opt_ (dB/μm)	FOM (°/dB)
532	1.75	4.37 × 10^–2^	2.24	0.78
1064	–1.02	2.81 × 10^–4^	1.44 × 10^–2^	70
1550	–0.43	6.27 × 10^–5^	3.22 × 10^–3^	132

## 5 Magnetic Domain Observation and Calculation

The domain structure of the 129-nm-thick Ce:YIG/GGG was observed using am MO Faraday effect (MOFE) microscope, described in the Methods Section (Figure 6a), at room temperature immediately after AC demagnetization of the sample. The FR difference between bright and dark domain regions was approximately ± 0.42°. An external OP field was applied using Helmholtz coils, enabling the field dependence of the domain structure to be correlated with the hysteresis loop; see Figure 6d.

**Figure 6:**
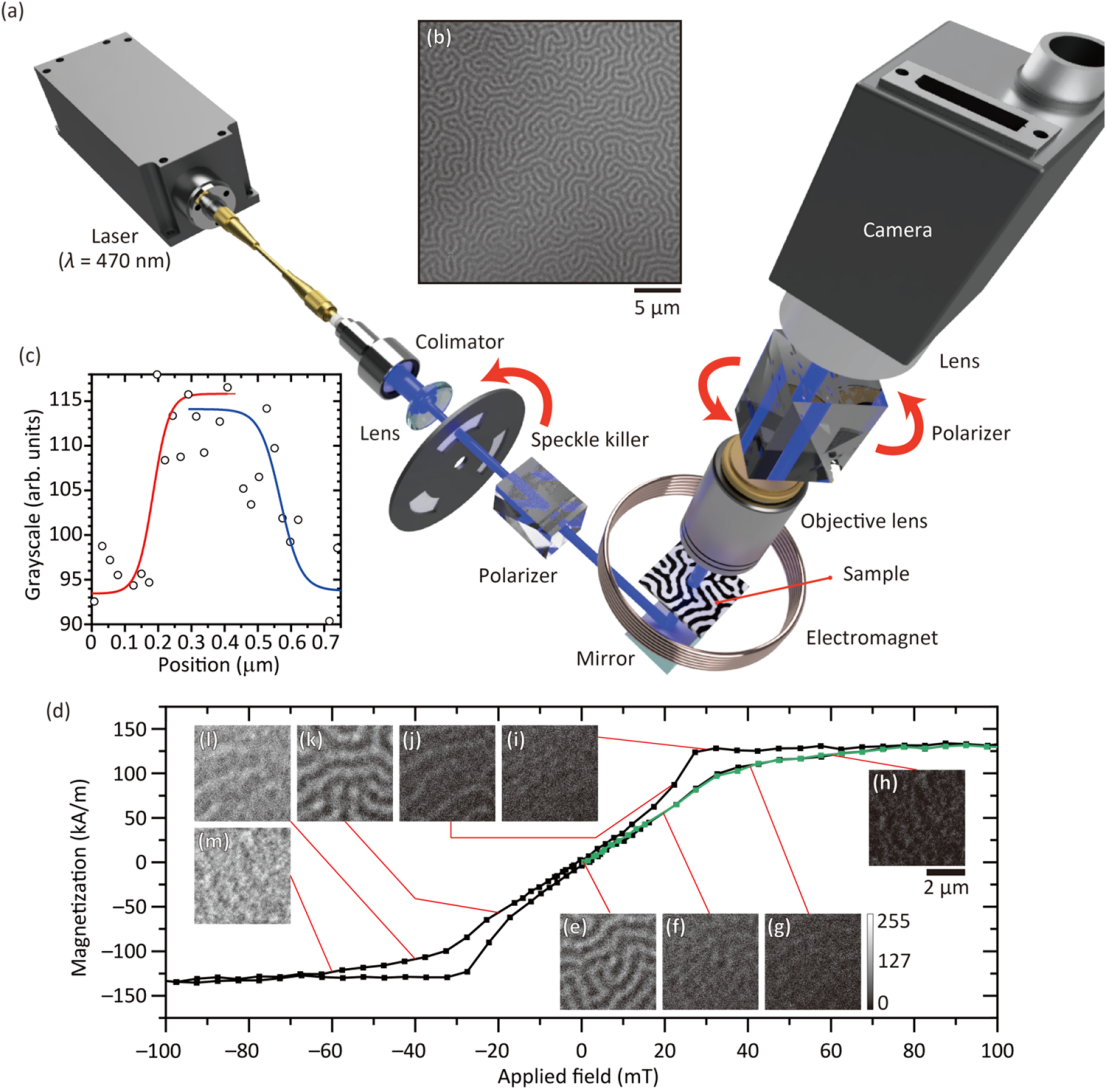
(a) Setup of the MOFE microscopy. The laser at a wavelength of 470 nm is incident to the sample through the fiber, collimator, speckle killer, and polarizer. The transmitted light was observed by the CMOS camera through the objective lens and polarizer. (b) The typical obtained MOFE image of Ce:YIG/GGG sample. (c) Line profiles across one magnetic domain fitted with Boltzmann-sigmoid functions. (d) OP magnetic hysteresis loop measured for Ce:YIG/GGG after subtraction of the paramagnetic component. The green line shows the initial magnetization curve. Magnetic domain images obtained by the MOFE microscopy under different applied magnetic fields: (e) 0, (f) 20, (g) 40, (h) 60, (i) 40, (j) 20, (k) −20, (l) −40, and (m) −60 mT.

Labyrinthine domains of Ce:YIG/GGG were present after demagnetization (Figure 6b) and their evolution under an OP field is shown by the green line in the magnetization curve from the demagnetized state to positive saturation. At zero field, the ratio of white to black regions was approximately equal, but field cycling caused domain expansion and a reduction in contrast. Ce:YIG/SGGG exhibited nearly identical behavior with 3% larger domains and DWs.

The magnetic domain width and domain wall (DW) width were extracted from the images, as exemplified in Figure 6b,c. The grayscale information was extracted along lines of pixels perpendicular to the longitudinal direction of a typical domain, as shown in Figure 6c, with a spatial resolution limit of approximately 23 nm imposed by the camera pixel pitch. A Boltzmann-sigmoid function fitting was performed to obtain the DW boundaries and widths (Methods Section). The example in Figure 6c has a domain width *w*_D_ of 219 ± 110 nm, yielding an average DW width of *w*_DW_ = 169 ± 67 nm. The half-period showed the total value of domain and DW width *w*_D+DW_ = 352 ± 18 nm. These values are based on the average and standard deviation of ten measurement points.

3D micromagnetic simulations were carried out by discretizing the film into a rectilinear mesh (see the Methods Section). The model dimensions were 5 μm × 5 μm × 130 nm, with mesh sizes of 10 nm × 10 nm × 10 nm cubic cells, and the model was solved using parallel computing on a supercomputer. Materials parameters were an anisotropy energy (excluding MS anisotropy) *K*_U_ – *K*_MS_ = 26.4 kJ/m^3^, a saturation magnetization *M*_s_ = 135 kA/m, and an exchange coupling constant *A*_ex_ of 4 pJ/m. The value of *A*_ex_ was optimized to fit the domain half-period observed in experiments (detailed procedures are described in ref 59) and is similar to reported values for YIG (3.5 pJ/m),^60^ Ca_1.0_Y_2.0_Ge_0.9_Fe_4.1_O_12_ (3.5 pJ/m),^61^ Ca_1.4_Y_1.6_Ge_1.4_Fe_3.6_O_12_ (1.5 pJ/m),^62^ and Ce_1_Y_2_Fe_5_O_12_ (1.2 pJ/m).^34^ Figure 7 shows the top and cross-sectional views of the relaxed structure. The DWs have a mixed character with Néel configuration near the film surfaces (i.e., IP moments to reduce stray field) but exhibit Bloch wall-like rotation in the middle of the thickness. Such twisted domain walls have been reported elsewhere.^63–65^ The DW width varies through the film thickness but is around 50 ± 20 nm, smaller than that obtained from MOFE. The domain half-period is 344 ± 55 nm, similar to that determined from MOFE of 352 ± 18 nm. This analysis revealed nonuniform magnetization states through the film thickness at DWs, explaining the wide DW boundaries in the MOFE images obtained using transmitted light.

**Figure 7:**
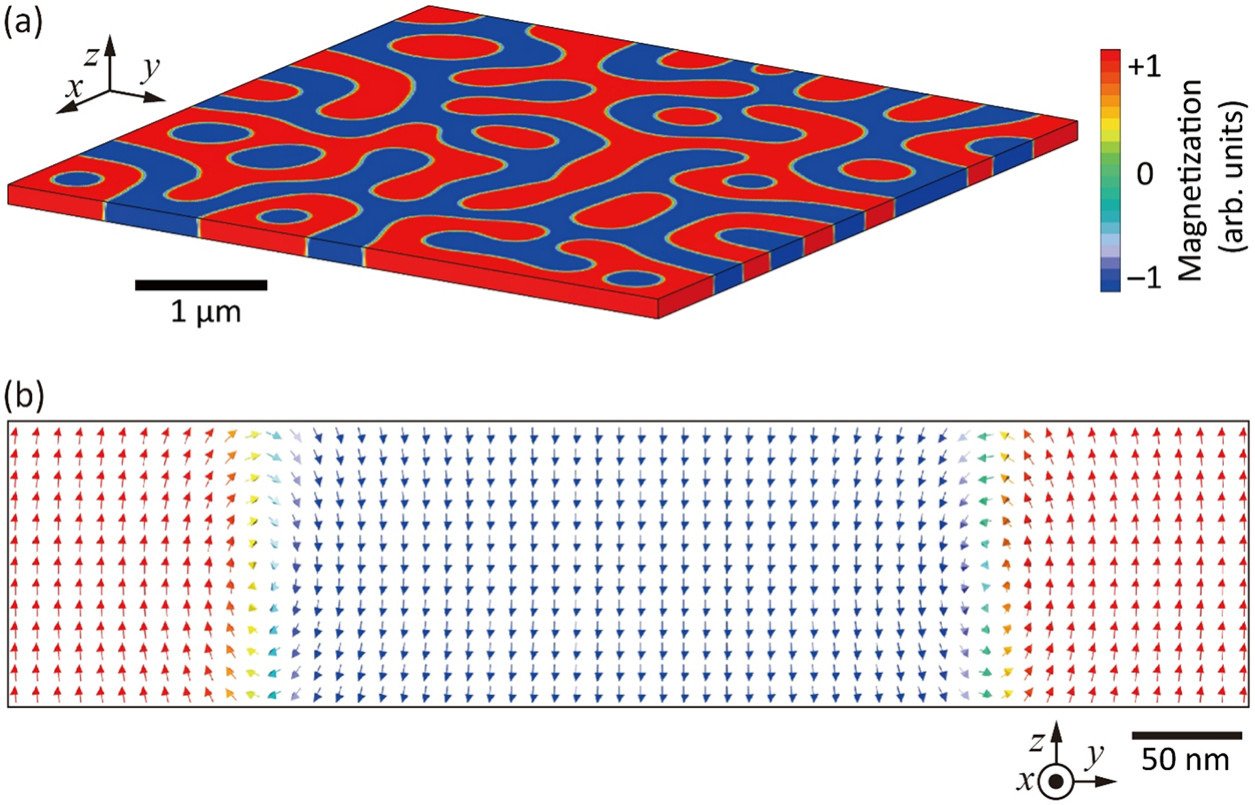
(a) Oblique view of the calculated magnetic domain pattern using a micromagnetic simulation. The color scale represents magnetization in the *z*-direction. (b) Cross-sectional views of magnetic domains and DWs. Arrows indicate magnetization directions, while the color represents the magnitude of the OP component of magnetization.

The width of the domains and DWs were compared to the analytical two-dimensional (2D) model of Kooy and Enz^66^ for a film that forms stripe domains. The film thickness *t* = 130 nm, magnetization *M*_s_ = 135 kA/m, and PMA excluding MS anisotropy *K*_U_ – *K*_MS_ = 26.4 kJ/m^3^. Assuming the DW has Néel character, the total energy of the domain structure *K*_D+DW_, which includes demagnetizing energy *K*_D_ and DW energy *K*_DW_, and the DW width *w*_DW_ are given by^65–67^

2 $$K_{D+\mathrm{DW}}=K_{D}+K_{\mathrm{DW}}$$

$$K_{D}=\frac{32M_{s}^{2}w_{D}}{\pi^{2}}\overset{21}{\underset{n=\mathrm{odd}}{\sum }}\frac{1}{n^{3}}\frac{\sin\;h\left(n\pi g\right)}{\sin\;h\left(n\pi g\right)+\sqrt{\mu }\cos\;h\left(n\pi g\right)}$$ 3

4 $$K_{\mathrm{DW}}=4\sqrt{A_{\mathrm{ex}}K_{U}}\frac{t}{w_{D}}$$

5 $$w_{\mathrm{DW}}=\pi \left(\sqrt{\frac{A_{\mathrm{ex}}}{K_{U}}}\right)$$

Here, *g* = (*t*/2*w*_D_)√μ, permeability μ is given by μ = 1 + (2π*M*_*s*_/*K*_U_), and *n* is an integer. The exchange constant is *A*_ex_ = 4 pJ/m. For a Bloch-type DW, the anisotropy energy *K*_U_ is replaced by *K*_U_ + 2π*M*_s_^2^ in the expression for *K*_DW_. The obtained Néel (Bloch) DWs showed a *w*_DW_ of 51 nm (39 nm) and a domain half-period *w*_D+DW_ of 364 nm (485 nm) with *w*_D_ = 313 nm (446 nm) from minimizing *K*_D+DW_. These values are summarized in Table S1. Therefore, the domain half-period and width of the Néel wall give a better fit to the experiment and that from 3D micromagnetic simulations, respectively, compared to the Bloch DW model.

## 6 Conclusion

We epitaxially grew Ce:YIG films on garnet substrates using IBS and demonstrated strain-independent PMA through MT anisotropy. The samples exhibited both enhanced Faraday rotation and robust PMA with submicrometer magnetic domains, independent of the substrate strain state. Detailed anisotropy analysis revealed that MT (growth-induced) anisotropy dominates over ME contributions, with a magnitude of up to ∼30 kJ/m^3^, sufficient to overcome the MS energy and achieve PMA regardless of tensile or compressive lattice mismatch strain. The labyrinth domain size in Ce:YIG films, observed using our constructed MOFE microscope, was approximately 352 ± 18 nm at remanence. Superior MO performance was achieved with a Faraday rotation of −1.05°/μm and figure of merit of 74.7°/dB at 1064 nm wavelength. The Ce:YIG magnetization can be switched by OP fields of 70 mT, making this a promising material for integrated MO devices. This work establishes a new paradigm for engineering magnetic anisotropy in garnet films through growth-induced anisotropy, transcending conventional strain engineering limitations and opening pathways for advanced MO applications.

## 7 Methods

### 7.1 Sample Deposition

The Ce:YIG film was epitaxially grown on (111)-oriented 10 mm × 10 mm GGG and SGGG substrates in the same batch. The substrates were double-side polished for optical and MO characterization, without antireflection coatings. The substrates were cleaned using isopropanol (IPA), acetone, and deionized (DI) water with sonication. The film was deposited using an RF-IBS system (RMTec, RM17-0010) with a base pressure of 4 × 10^–5^ Pa. During deposition, 8 sccm of O_2_ was introduced. The working pressure was 3 × 10^–2^ Pa. The substrates were heated to 810 ± 30 °C using a lamp heater set to 900 °C according to previous reports^26,27,33,44^ and rotated at 4.3 rpm to ensure film uniformity. A 4-in. diameter sintered Ce_1.0_Y_2.5_Fe_5_O_*x*_ (Furuuchi Kagaku Co.) target was used. The ion beam voltage and current were 800 V and 36 mA, respectively. 10 and 14 sccm of Ar flowed to the ion gun and a low-frequency neutralizer (LFN), respectively. The ion beam accelerator voltage was 160 V, and the RF power was 75 W. The deposition rate for Ce:YIG was 1.54 nm/min. This deposition condition is the same as in the previous study on Ce:YIG growth on a YAG substrate.^33^

### 7.2 Thickness Measurement

The thickness of the Ce:YIG film, measured using a stylus profilometer (KLA Tenscor α-Step IQ), was 129 ± 4.5 nm. The error represents the standard deviation of nine repeated measurements. The measurements were taken with a stylus force of 12 mg over a length of 500 μm at a speed of 2 μm/s, and a sampling rate of 50 Hz was used.

### 7.3 XRD Measurement

The crystalline structure was characterized using an XRD. A Cu Kα_1_ (wavelength λ = 0.15418 nm) radiation source was used with X-ray power of 3 kW. The optical receiving system consisted of a 5.0° Soller slit and high-resolution parallel beam Ge (220) × 2 analyzer. A one-dimensional X-ray detector (D/teX Ultra 250) was used for detection. (110) order peaks were probed with a Bruker D8 Discover GADDS equipped with a Co Kα_1_ (λ = 0.1790 nm) radiation source, 1/4 Eulerian cradle, and a Vantec-2000 area detector.

### 7.4 Determination of Lattice Constant and Angle

The crystal axis length *A*_CS_ and crystal angles θ_cs_ corresponding to the (444) and (1̅1̅2) orientations can be expressed using the relationship between the interplanar spacing of reciprocal lattice vectors *q*_*x*_ = *d*_1̅1̅2_^–1^ and *q*_*z*_ = *d*_444_^–1^ as follows^49^

6 $$\frac{1}{{d_{\mathrm{1̅}\mathrm{1̅}2}}^{2}}=6\frac{\sin^{2}\theta_{\mathrm{CS}}-\left(\cos^{2}\theta_{\mathrm{CS}}-\cos\theta_{\mathrm{CS}}\right)}{A^{2}\left(1-3\cos^{2}\theta_{\mathrm{CS}}+2\cos^{3}\theta_{\mathrm{CS}}\right)}$$

7 $$\frac{1}{{d_{444}}^{2}}=6\frac{\sin^{2}\theta_{\mathrm{CS}}+2\left(\cos^{2}\theta_{\mathrm{CS}}-\cos\theta_{\mathrm{CS}}\right)}{A^{2}\left(1-3\cos^{2}\theta_{\mathrm{CS}}+2\cos^{3}\theta_{\mathrm{CS}}\right)}$$

### 7.5 TEM Images

TEM and elemental mapping of the cross-sectional images were performed. For TEM measurements, the samples were diced into 5 mm squares using a dicer (Disco, DAD321) and then thinned into films using an ion slicer (JEOL EM-09100IS).

### 7.6 XPS Measurement

XPS (Shimadzu Kratos Axis Ultra) was used to investigate the electronic binding states of the film. The X-ray source was monochromated Al Kα with an energy of 1486.69 eV. The X-ray output conditions were set to 15 kV, 10 mA, and 150 W for the voltage, current, and power, respectively. To minimize the effects of surface contamination and oxidation, the sample surfaces were etched for 5 s using 2 kV Ar sputtering prior to measurement. Charge correction after etching was performed using Y 3d doublet peaks, calibrated against the C 1s peak from the preetching spectra. The Ce^4+^/Ce^3+^ ratio for the Ce:YIG film was calculated from the fitted peak regions using the CasaXPS software.^68^

### 7.7 MO Characterization

MO measurements were conducted with an MO measurement system (JASCO, J-1700FK) utilizing rotating polarizer and polarization modulation methods. To minimize the temperature-dependent magnetization effects of Ce:YIG, the measurements were performed at 40 ± 0.5 °C controlled by a Peltier device. The incident light covered an area of 1 mm × 5 mm on the sample surface, with an applied magnetic field of ±500 mT during spectrum and loop measurements. Visible and NIR wavelength regions were detected using a photomultiplier and InGaAs-based optical detectors, respectively. The system achieved FR and FE resolutions of <0.01 and <0.1°, respectively. FR and FE values were defined as positive for clockwise rotation when viewed from the detector toward the source.^35,57^

### 7.8 Optical Transmissivity Measurement

Optical transmissivity was measured by using a spectrophotometer (Shimadzu UV-3150) with a double monochromator and randomly polarized lamp source. The scanning speed was set to “slow” mode, with a wavelength resolution of 5 nm. The irradiated spot diameter perpendicular to the sample surface was 2.2 mm.

### 7.9 MOFE Microscope

To observe magnetic domains, an MOFE microscope was constructed, as shown in Figure 6. A diode laser operating at 470 nm (Edmund Optics, MDL-III-470-200 mW-SMA) with a nominal output power of 200 mW was used as the light source, taking advantage of the larger FR (∼3.27°/μm) at this wavelength while maintaining sufficient transmissivity (57%), as confirmed in Figure 5a,b. The beam was shaped using a fiber collimator (Thorlabs, F810SMA-543) followed by a planoconvex lens positioned 20 mm from the collimator lens. To eliminate the speckle noise inherent to laser illumination, we implemented a speckle killer by attaching a tissue paper to a rotating optical chopper (NF, 5584A). The optical path incorporated Glan–Thompson prisms (Karl Lambrecht, MGT25B12) as polarizers and an analyzer (Karl Lambrecht, MGTYB15) mounted on an automated stage (Sigma Koki, SGSP-60YAW-OB). The transmitted light was detected by a CMOS camera (Basler, ace acA5472-17) through an imaging unit (Olympus, U-TV1X). Sample focusing was achieved using a mechanical stage (Chuo Precision Industrial, LS912-WS) for objective lens movement and an automated stage (Sigma Koki, SGSP20-20) for sample positioning. A perpendicular magnetic field of up to ± 60 mT was applied to the sample during observation using Helmholtz coils driven by a bipolar power supply (Kenwood, PD18-30A). A ring-shaped permanent magnet was used to apply magnetic fields of ± 60 mT, while the Helmholtz coils were employed for fields up to ± 50 mT due to setup limitations.

### 7.10 Magnetic Domain Profile Fitting and Width Determination

The grayscale information *G* of the measured magnetic domains was fitted by using the following procedure. The fitting was performed individually for each domain wall. First, we extracted data from one domain wall region and an adjacent domain region of similar length. These data were then fitted using the Boltzmann-sigmoid function

8 $$G=\frac{G_{1}-G_{2}}{1+e^{\left(x-x_{0}\right)/p}}+G_{2}$$

where *G*_1_ and *G*_2_ represent the maximum and minimum values of *G*, *x* denotes the position along the horizontal axis, *x*_0_ is the inflection point with maximum slope, and *p* represents the gradient. The boundary points between the DW and domain were determined as the positions *x* where the tangent line at the inflection point *x*_0_ intersects with *G*_1_ or *G*_2_.^69,70^

### 7.11 Simulation of the Magnetic Domain Structure

The thickness matched the experimental Ce:YIG value of 130 nm, while the area was chosen to be more than 10 times larger than the domain width to minimize computational artifacts. An energy minimization method was used to derive the magnetic domain states. The model was surrounded by empty cells (i.e., air) to allow stray fields to be calculated, with total dimensions of 40 μm × 40 μm × 40 μm. The sample region was meshed with 10 nm × 10 nm × 10 nm cubic cells, based on the expected characteristic length and DW width of 12 and 39 nm, respectively. Larger mesh sizes were used in the air region to reduce the computation time. Due to the large number of mesh elements (3.3 × 10^6^ for the sample region and 2.4 × 10^7^ total), parallel computing on a supercomputer was employed. The values of *M*_s_ and *K*_U_ used in the simulation were obtained from VSM measurements, and the value of *A*_ex_ was determined by fitting to the magnetic domain half-period observed by MOFE microscopy.

### 7.12 Supercomputer Specification

The calculations were performed using EXAMAG simulation software (Fujitsu, GUI version 2.3.d55, and solver versions 2.3.d39 and 0.9.0). Two supercomputer systems were employed. The first was AOBA-B at Tohoku University (TU), utilizing 128 cores and 256 GB of memory per node, with a computational performance of 4.096 TFlops based on AMD EPYC 7702 processors. The second was Fugaku at RIKEN, utilizing 144 cores and 96 GB of memory across three nodes, with a computational performance of 3.072 TFlops. The calculation time for one magnetic domain pattern was 7 and 2 h on each system, respectively.

### 7.13 Uncertainty Estimation

Unless otherwise stated, uncertainties reported throughout this article represent the following: Uncertainties in film thickness, saturation magnetization, anisotropy energy, anisotropy field, and Faraday rotation values are primarily derived from the standard deviation of the film thickness measurements (nine measurement points). Magnetic domain and domain wall width uncertainties represent the standard deviation from ten measurement points. Deposition temperature uncertainty represents the maximum deviation from the actual substrate temperature, arising from calibration based on a reference location rather than direct substrate temperature measurement. In contrast, the measurement temperature uncertainties indicate the maximum range of fluctuations observed during the process. Crystal angle uncertainties in XRD measurements represent the instrumental precision of the diffractometer. Coercivity uncertainties represent the difference in coercivity values obtained from positive and negative field branches of the hysteresis loops.

## Supplementary Material



## Notes

Cross-sectional TEM-HAADF images of Ce:YIG on GGG and SGGG substrates; selected-area electron diffraction patterns; Faraday rotation and ellipticity spectra and loops at multiple wavelengths; transmissivity spectra; refractive index and extinction coefficient spectra; domain width measurements comparison table between the experiment and simulations; and derivation of magnetostriction constant assuming low magnetoelastic anisotropy contribution (PDF)
