# Supplementary material for: Magnetotaxial Perpendicular Magnetic Anisotropy and Enhanced Faraday Rotation in Ion Beam Sputtered Cerium-Substituted Yttrium Iron Garnet
Source: ACS Appl Opt Mater. 2025 Nov 25;3(12):2923–34. doi: 10.1021/acsaom.5c00496 (PMC12751105; doi:10.1021/acsaom.5c00496)
Supplement: Supplementary file 1 [file ot5c00496_si_001.pdf]

Supporting Information

Magnetotaxial perpendicular magnetic anisotropy and enhanced Faraday rotation in ion beam sputtered cerium-substituted yttrium iron garnet

Taichi Goto,[1,*] Takumi Koguchi,[1,2,3] Yuki Yoshihara,[1,2,3] Hibiki Miyashita,[1,2] Kanta Mori,[1,2] Toshiaki Watanabe,[4] Allison C. Kaczmarek,[5] Pang Boey Lim,[3] Mitsuteru Inoue,[1] Caroline A. Ross,[5] Kazushi Ishiyama[1]

[1] Research Institute of Electrical Communication, Tohoku University, 2-1-1 Katahira, Aoba, Sendai, Miyagi 980-8577, Japan.

[2] Graduate School of Engineering, Tohoku University, 6-6 Aramaki, Aoba, Sendai, Miyagi 980-8579, Japan.

[3] Toyohashi University of Technology, 1-1 Hibarigaoka, Tempaku, Toyohashi, Aichi 441-8580, Japan.

[4] Shin-Etsu Chemical Co., Ltd., 2-13-1 Isobe, Annaka, Gunma 379-0195, Japan.

[5] Massachusetts Institute of Technology, 77 Massachusetts Avenue, Cambridge, Massachusetts 02139, United States.

Contact Information

* Corresponding author: Taichi Goto, (Dr., Prof.)

Phone: +81-22-217-5489

Email: taichi.goto.a6@tohoku.ac.jp

Address: Research Institute of Electrical Communication, Tohoku University, 2-1-1 Katahira, Aoba, Sendai, Miyagi 980-8577, Japan

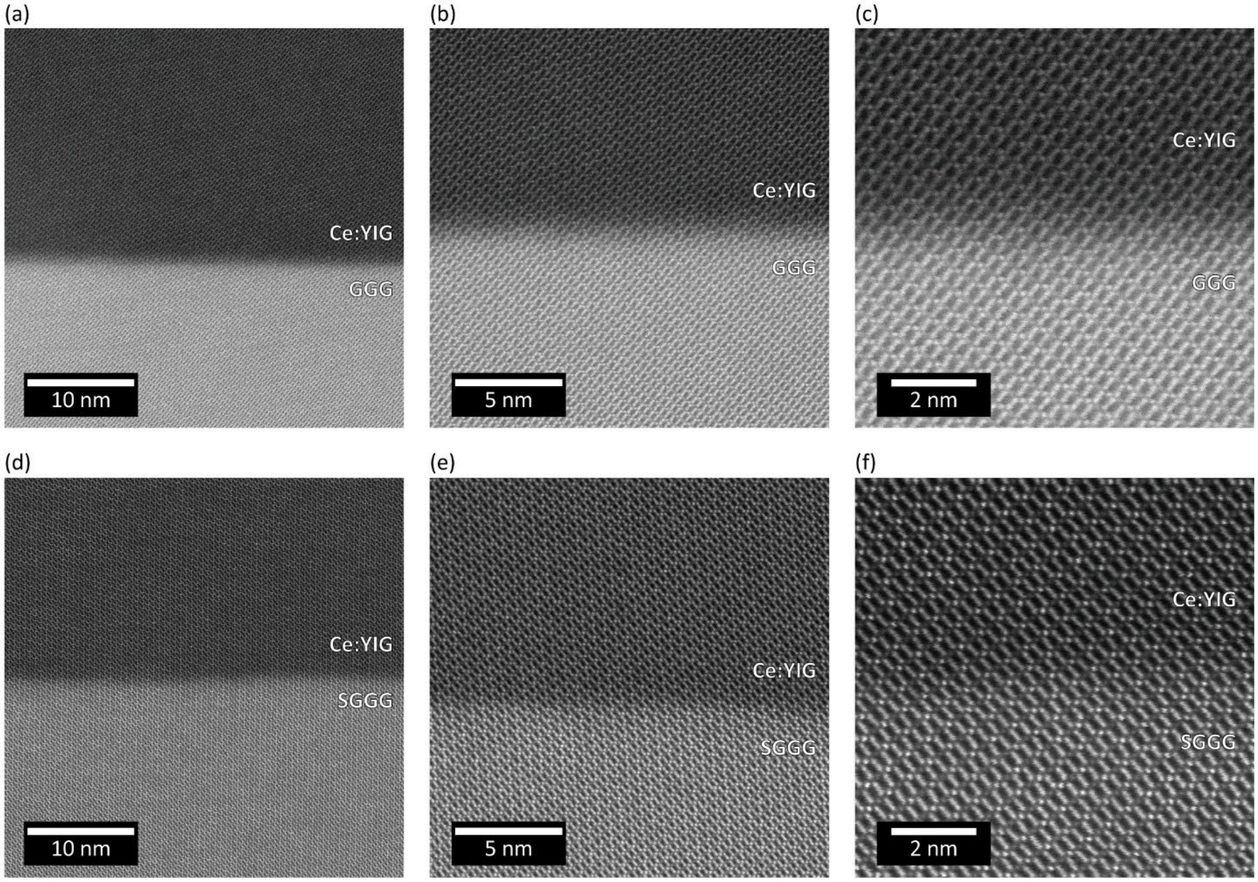

Figure S1. Cross-sectional transmission electron microscopy (TEM) high-angle annular dark-field (HAADF) images of Ce:YIG on GGG (a,b,c) and SGGG (d,e,f).

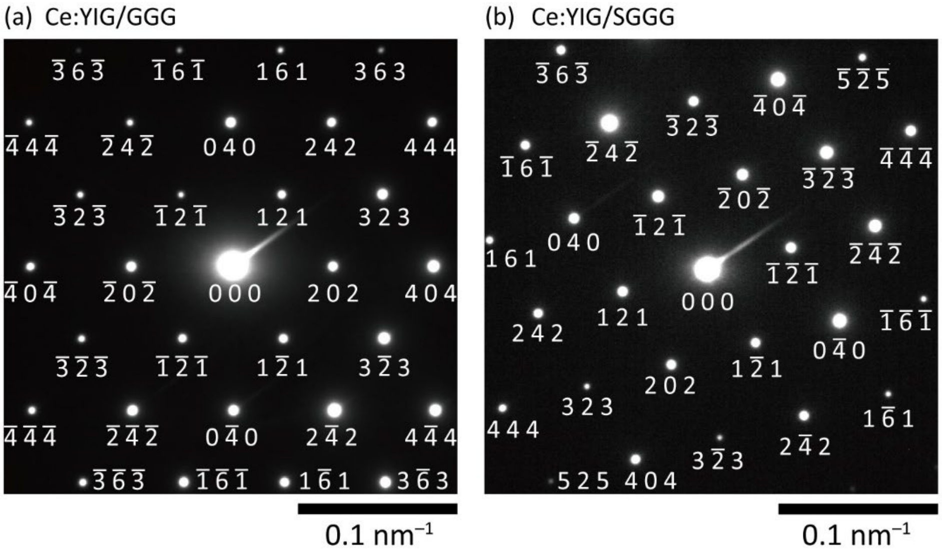

Figure S2. Selected-area electron diffraction (SAD) patterns of Ce:YIG on GGG (a) and SGGG (b).

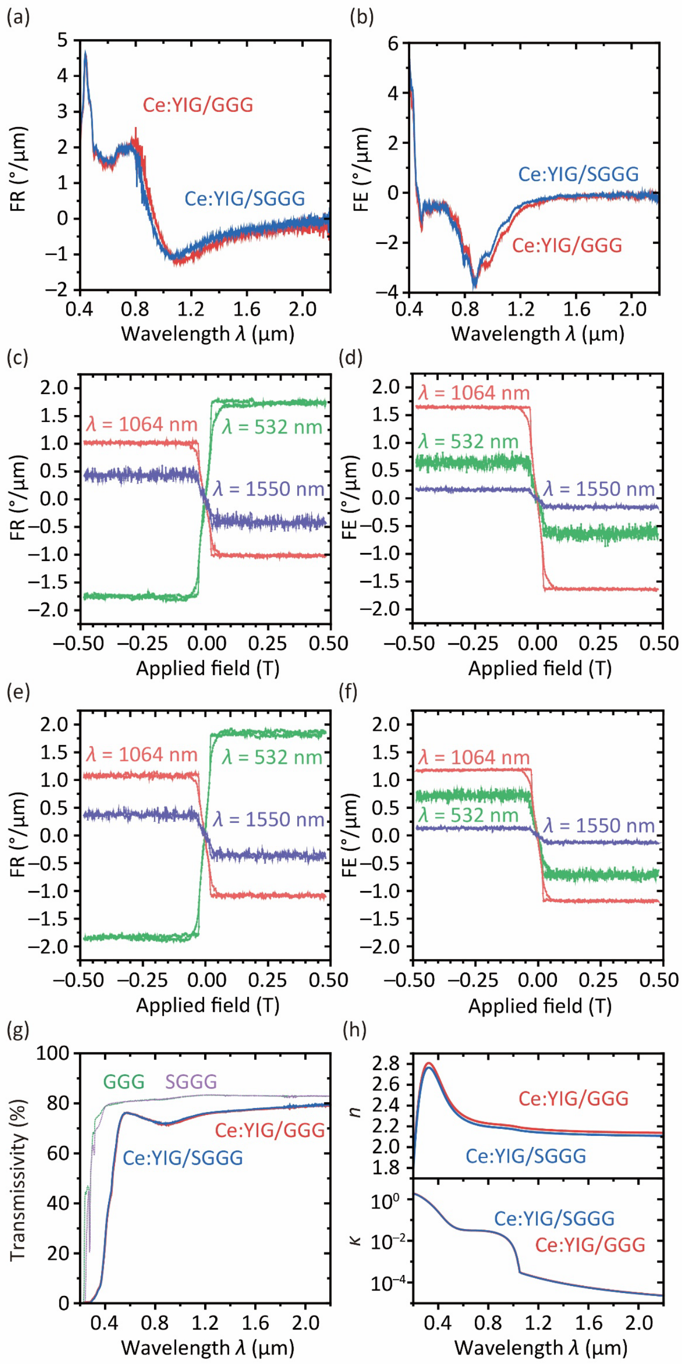

Figure S3. (a) Faraday rotation (FR) and (b) Faraday ellipticity (FE) spectra of Ce:YIG/GGG and Ce:YIG/SGGG. (c) FR and (d) FE loops of Ce:YIG/GGG, and (e) FR and (f) FE loops of Ce:YIG/SGGG measured at wavelengths of 532 nm, 1064 nm, and 1550 nm. All FR and FE data were measured after subtraction of the substrate contributions. (g) Transmissivity spectra of Ce:YIG/GGG, Ce:YIG/SGGG (including substrate contributions), GGG, and SGGG. (h) Refractive index n and extinction coefficient κ spectra of extracted from transmission spectra.

Table S1. Domain width w_D, domain wall (DW) width w_{DW}, and domain half-period w_{D+DW} obtained by experiment, three-dimensional (3D) simulation, and two-dimensional (2D) analysis.

	Experiment (nm)	3D simulation (nm)	2D analysis	
			Néel (nm)	Bloch (nm)
w_D	219±110	294±20	313	446
w_{DW}	169±67	50±20	51	39
w_{D+DW}	352±18	344±55	364	485

Note S1. Derivation of the magnetostriction constant with assuming low contribution of magnetoelastic anisotropy on total magnetic anisotropy energy.

We assume the same K_{MT} for both films and allow λ_{111} to vary from the extrapolated value. Here we show the detailed derivation of λ_{111} based on the total magnetic anisotropy energy with assuming the saturation magnetization of Ce:YIG on GGG and SGGG substrates are the same.

$$\begin{cases} K_{\mathrm{U}} = K_{\mathrm{MC}} + K_{\mathrm{ME}} + K_{\mathrm{MS}} + K_{\mathrm{MT}} \\ K_{\mathrm{U,GGG}} = K_{\mathrm{MC}} + \frac{9}{4}\lambda_{111}c_{44}\left(\frac{\pi}{2} - \theta_{\mathrm{CS,GGG}}\right) + K_{\mathrm{MS}} + K_{\mathrm{MT}} \\ K_{\mathrm{U,SGGG}} = K_{\mathrm{MC}} + \frac{9}{4}\lambda_{111}c_{44}\left(\frac{\pi}{2} - \theta_{\mathrm{CS,SGGG}}\right) + K_{\mathrm{MS}} + K_{\mathrm{MT}} \end{cases} \tag{S1}$$

We eliminated the K_{MC}, K_{MS}, and K_{MT}:

$$K_{\mathrm{U,GGG}} - \frac{9}{4}\lambda_{111}c_{44}\left(\frac{\pi}{2} - \theta_{\mathrm{CS,GGG}}\right) = K_{\mathrm{U,SGGG}} - \frac{9}{4}\lambda_{111}c_{44}\left(\frac{\pi}{2} - \theta_{\mathrm{CS,SGGG}}\right) \tag{S2}$$

We rearranged the equation to isolate the λ_{111} term on the left-hand side:

$$\lambda_{111} = \frac{4}{9}\frac{K_{\mathrm{U,GGG}} - K_{\mathrm{U,SGGG}}}{c_{44}\left(\theta_{\mathrm{CS,SGGG}} - \theta_{\mathrm{CS,GGG}}\right)} \tag{S3}$$
